# Development of a Data-Based Method for Predicting Nursing Workload in an Acute Care Hospital: Methodological Study

**DOI:** 10.2196/66667

**Published:** 2025-09-16

**Authors:** Mark McMahon, Sylvie Plate, Tobias Herz, Gabi Brenner, Michael Kleinknecht-Dolf, Michael Krauthammer

**Affiliations:** 1 Department of Quantitative Biomedicine, University of Zürich, Schmelzbergstrasse 26, Zürich, 8006, Switzerland, 41 446356631; 2 Biomedical Informatics DFL, University Hospital Zürich, Schmelzbergstrasse 26, Zürich, 8006, Switzerland; 3 Directorate of Nursing and Allied Health Care Professionals, University Hospital Zürich, Zürich, Switzerland

**Keywords:** nursing staff, hospital, workload, forecasting, linear models, statistical modeling, machine learning, nurse, workload, acute care, staff, machine learning, retrospective, observational, nursing workload, data, prediction, predictive

## Abstract

**Background:**

Determining effective nurse staffing levels is crucial for ensuring quality patient care and operational efficiency within hospitals. Traditional workload prediction methods often rely on professional judgment or simple volume-based approaches, which can be inaccurate. Machine learning offers a promising avenue for more data-driven and precise predictions, by using historical nursing workload data to forecast future patient care requirements, which could help with staff planning while also improving patient outcomes and nurse well-being.

**Objective:**

This methodological study aimed to use nursing activity data, specifically LEP (Leistungserfassung in der Pflege; “documentation of nursing activities”), to predict the future workload requirements using machine learning techniques.

**Methods:**

We conducted a retrospective observational study at the University Hospital of Zürich, using nursing workload data for inpatients across eight wards, collected between 2017 and 2021. Data were transformed to represent nursing workload per ward and shift, with 3 shifts per day. Variables used in modeling included historical workload trends, patient characteristics, and upcoming operations. Machine learning models, including linear regression variants and tree-based methods (Random Forest and XGBoost), were trained and tested on this dataset to predict workload 72 hours in advance, on a shift-by-shift basis. Model performance was assessed using mean absolute error and mean absolute percentage error, and results were compared against a baseline of assuming no change in workload from the time of prediction. Prediction accuracy was further evaluated by categorizing future workload changes into decreased, similar, or increased workload relative to current shift levels.

**Results:**

Our findings demonstrate that machine learning models consistently outperform the baseline across all wards. The best-performing model was the lasso regression model, which achieved an average improvement in accuracy of 25.0% compared to the baseline. When used to predict upcoming changes in workload levels, the model achieved strong classification performance, giving an average area under the receiver operating characteristic curve of 0.79 and precision values between 66.2% and 75.3%. Crucially, the model severely misclassified—predicting an upcoming increase as a decrease, and vice versa—in just 0.17% of cases, highlighting potential reliability for using the model in practice. Key variables identified as important for predictions include historical shift workload averages and overall ward workload trends.

**Conclusions:**

This study suggests the potential of machine learning to enhance nurse workload prediction, while highlighting the need for refinement. Limitations due to potential discrepancies between recorded nursing activities and the actual workload highlight the need for further investigation into data quality. To maximize impact, future research should focus on: (1) using more diverse data, (2) more advanced machine learning architecture that performs time-series modeling, (3) addressing data quality concerns, and (4) conducting controlled trials for real-world evaluation.

## Introduction

### Overview

Determining the correct nurse staffing levels for a hospital shift is crucial for ensuring both quality of patient care and operational efficiency [1]. Due to the increasing scarcity of resources and the intensification of the use of these resources, as well as the goals of preserving patient safety and job attractiveness, there has long been an interest in effective staffing and scheduling of nurses. An essential step in creating reliable time-based personnel schedules is accurately estimating the expected patient-related nursing workload [2]. Consequently, the development of a corresponding model or tool for clinical practice is of great interest.

### Background

In recent years, various methodologies have been used for estimating upcoming patient-related nursing workload. These include professional judgment, simple volume-based methods such as nurse-to-patient ratios, and more detailed patient classification systems where patients are grouped according to their nursing needs [3,4]. Further to these approaches, there are also timed-task approaches which rely on a detailed nursing care plan, where nursing activities for each patient are reported [5], and used to estimate upcoming nursing requirements.

Despite extensive research, systematic reviews have consistently highlighted that many of these tools fail to provide robust, scalable, and universally applicable methods for estimating workload in dynamic clinical settings. A key limitation is that these models often rely on static assumptions rather than continuously adapting to the evolving needs of patients and hospital units. As a result, there is no clear consensus and little basis to prefer one tool over another [3].

In order to adequately capture nursing work, it is important to define what nursing is and which measures are included as a basis for their development [6]. Our study is based on the definition developed for professional nursing in Switzerland [7,8]:

It is the mission of professional nursing to preserve and promote health and to support individuals dealing with the impact of disease and treatment. In addition, it is the goal of nursing to achieve the best possible treatment outcomes and care as well as the best possible quality of life for the patient.

Nursing workload data can be extracted out of the electronic health records (EHR) of the patient, where nurses document the patient-related activities. Using the detailed information captured, which includes specifics on nursing activities and the time associated with them, offers a promising avenue for enhancing the accuracy of nurse workload predictions [9,10]. The development of a model that uses these data could enhance how nurse staffing is approached, leading to better alignment between daily staffing needs and actual patient care demands [11].

Machine learning is a rapidly advancing field that offers the ability to uncover past patterns and make predictions based on extensive data. Machine learning tools have been applied across a large range of contexts, and they have proven successful in medical settings [11]. In the context of nursing workload estimation, there are initial publications on experiences with the use of historical workload data for workload estimation tools, but while initial studies indicate potential to address the shortcomings of traditional methods, evidence remains inconclusive [10,12].

### Previous Work

While recent studies highlight the potential usefulness of workload forecasting [13], most remain exploratory in nature and do not provide actionable shift-level predictions (for a more detailed overview, see Multimedia Appendix 1). Some studies focus on simply identifying workload predictors rather than developing full predictive models [14], while others investigate alternative data collection methods, such as video-based workload estimation [15,16]. In addition, many studies classify current workload levels rather than predicting future nursing demands, making them less useful for proactive staff planning [17]. A recent study showed good performance next-shift workload prediction in an intensive care unit setting [12], but this was limited in scope and generalizability, and the short forecast horizon makes it impractical for use in hospital staff planning.

### Objective

The objective of this methodological study is to use machine learning to accurately predict future nursing workloads for a ward. Specifically, the focus is on forecasting patient-related workload requirements for a shift, 72 hours (or 9 shifts) ahead of time, where accurate predictions would provide valuable support for staff planning.

### Proposed Approach

Our approach aims to move beyond the aforementioned limitations by developing a predictive model that uses historical nursing workload data to forecast future demands. By using machine learning techniques with EHR-derived workload data, we shift from static, rule-based approaches to a data-driven framework. This enables more precise nurse staffing predictions, enhances operational efficiency, and supports improved patient care by proactively anticipating workload fluctuations.

## Methods

### Design and Methodology

To achieve the study’s aim, a retrospective observational study design was used. The methodology involved a retrospective chart review, utilizing nursing workload data, specifically LEP (Leistungserfassung in der Pflege; “documentation of nursing activities”) data [18,19].

### Setting

This study, conducted at the University Hospital Zürich, used nursing workload data derived from nursing care activities recorded for patients hospitalized between 2017 and 2021. The hospital comprises multiple specialized wards, providing comprehensive care across various disciplines. We included data from inpatients across 8 wards, as detailed in Table 1. Each ward operates with 3 daily shifts: the night shift (midnight - 7:59 AM), the early shift (8 AM- 3:59 PM), and the late shift (4 PM - 11:59 PM). The selection of these wards was based on maximizing the number of cases per ward, ensuring homogeneity within patient groups, and minimizing the potential impact of database changes during the data collection period (2017‐2021).

**Table 1.** Summary statistics of patients and wards based on raw LEP data.

Ward	Ward type	Number of Patients^a^	Average age (years)	Percent female (%)	Total interventions	Average length of each intervention (minutes)
Overall	All wards	28,930	56.6	50.6	11,226,067	4.9
Ward A	Neurology	6858	61.9	47.1	1,385,717	5.3
Ward B	Neurology	5147	65.3	42.3	1,175,214	5.9
Ward C	Neurology IMCU^b^	4479	60.4	45.5	1,173,311	4.2
Ward D	Urology	5766	58.3	23.7	2,027,421	4.4
Ward E	Maternity	6733	32.5	97.1^c^	1,731,118	4.6
Ward F	Neurosurgery	7170	58.5	47.8	1,658,616	4.9
Ward G	Neurosurgery	4589	58.3	47.4	838,631	4.8
Ward H	Neurosurgery IMCU^b^	2873	67.3	41.6	1,236,039	5.6

a Patients can occupy multiple wards during a stay or across visits, so they are counted in each ward individually, but only once overall. As a result, the total patient count is lower than the sum of the individual ward counts.

b IMCU: intermediate care unit.

c Some newborns (including males) also receive nursing care in the maternity ward.

d Actual ward names have been removed due to data privacy.

### Data Collection

The documentation of nursing activities, used in this study as workload data, is contained in the patients’ electronic health record within the hospitals' clinical information system, KISIM (Klinikinformationssystem, clinical information system). KISIM is a Swiss hospital information system that assists staff with the planning and execution of hospital processes. The hospital’s Information Technology team, within the Directorate of Research and Education, extracted and provided this data. In addition to workload data, we also received data relating to patient medication and planned operations.

In this study, nursing workload is defined as the time spent on direct patient nursing activities in the presence of the patient, family, or both, and indirect nursing activities performed on behalf of the patient, with LEP as the documentation tool and data source.

LEP is a standardized nursing activity classification system that has been designed to record detailed data on nursing workloads and care given to patients and is used in over 1000 institutions in Switzerland, Germany, Austria, and Italy. LEP records direct and indirect patient-related nursing activities by case, day, and ward. Developed through participant observations and interviews with Swiss hospital nurses, LEP ensures that its categories accurately reflect professional nursing practices in the region. Each activity is assigned a time value based on how long a trained, experienced nurse would typically take to complete it, while maintaining professional quality standards [18,19]. These default times are re-evaluated regularly, and nurses are able to overwrite the default times for the majority of nursing activities to reflect the actual time required for a particular activity [20]. Today, LEP is implemented in many software applications, such as the KISIM application at the University Hospital of Zurich, which is used as the data source for this study [20].

### Data Transformation

**Figure 1.**
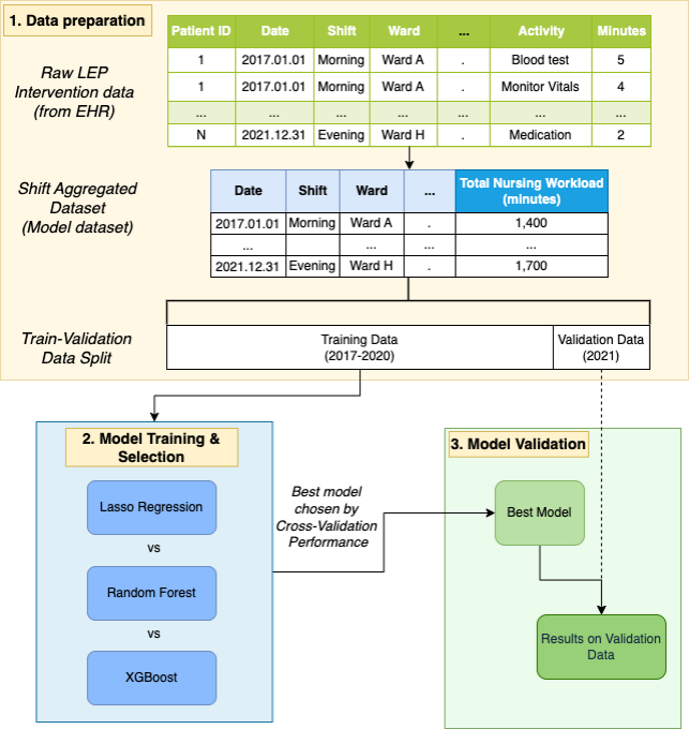
Overview of the project pipeline for nursing workload prediction. Raw interventional data (source: LEP Intervention Data) are collected and aggregated at the shift level to create a dataset of total nursing workload (min) for each ward and shift combination, to be used in the modeling phase. This dataset is split into training (2017‐2020) and validation (2021) data. Three machine learning models (lasso regression, random forest, and XGBoost) are trained and evaluated using time-series cross-validation. The best-performing model is selected and validated on the holdout dataset to assess its generalizability*.* EHR: electronic health records; LEP: Leistungserfassung in der Pflege (documentation of nursing activities).

Figure 1 shows our project pipeline for predicting upcoming nursing workload, from data extraction and preparation through to model training and validation. The “Data Preparation” section describes our process of transforming the low-level LEP data, expressed in minutes of nursing time provided, into a shift level dataset, so as to get the recorded workload per ward for each shift. During plausibility checks, we identified instances where some patients had workload entries for 2 different wards during the same shift. This was considered feasible, as patient transfers between wards are common, and while some cases may have been due to data quality problems, overall these instances represented a minor portion of the data and were therefore kept in the dataset. After transformation and feature creation, no missing data was identified, allowing us to use the full dataset for analysis.

### Outcome Variable

Our outcome variable was the patient-related nursing workload (ie, the documented workload in LEP) needed for each ward per shift. Using historical data, we predicted the nursing workload 72 hours (9 shifts/3 d) before the start of the shift and then compared these predictions with the actual workload documented in the LEP system. This retrospective comparison enabled us to assess the accuracy of our predictions.

In this paper, we refer to a “prediction window,” by which we mean the time between the point at which the prediction is made and the shift for which the prediction is made. The prediction window is therefore 9 shifts (equivalent to 72 h or 3 d) into the future, from the time of prediction.

### Variables Used in Modeling

We derived a set of variables which were to be used during modeling (Table 2), using the full data range of available data sources to create the feature set. This set included summaries of the historic trends, such as shift mean and ward mean, which represent the average workload required for that shift or ward in the past. Descriptive features included the type of shift (night, early, or late shift), the specific ward (Ward A to Ward H), the month, and the day of the week. In addition, patient-specific data points such as the number of patients on the ward at the time of prediction, the total number of medications given to patients on the ward, the gender ratio (percentage of male patients), the average age of the patients, and the number of upcoming patient operations were also included.

**Table 2.** Variables used in the modeling.

Variable name	Type	Description
Ward	Categorical	Which of the eight hospital wards we are predicting for: Ward A, …, Ward H
Shift type	Categorical	Shift we are predicting for: night, early, or late shift
Month	Cyclical	—^a^
Day of week	Cyclical	—^a^
Shift mean (Overall)	Numeric	The average nursing workload required for the given shift type, calculated using past data
Ward mean (Overall)	Numeric	The average nursing workload required for the given ward, calculated using past data
Shift mean (Last month)	Numeric	The average nursing workload required for the given shift type, calculated using the most recent month of data
Shift mean (Last 9 shifts)	Numeric	The average nursing workload required for the given shift type, calculated using the most recent nine shifts of data
Ward mean (Last 9 shifts)	Numeric	The average nursing workload required for the given ward, calculated using the most recent nine shifts of data
Number of patients	Numeric	Number of patients on the ward at the time of prediction
Previous number of patients	Numeric	Number of patients on the ward *i* shifts ago, for i=1, 2, 3
Previous workload	Numeric	The nursing workload required at that ward *i* shifts ago, for i=1, …, 9
Number of medications given	Numeric	Total number of medication types given to patients in the ward at time of prediction
Proportion patients male	Numeric	Percentage of patients on the ward at the time of prediction who are male
Average age	Numeric	Average age of all patients on the ward at the time of prediction
Upcoming operations	Numeric	Number of operations that patients on the ward have planned in the prediction window

a Not applicable.

Features based on past trends, such as Shift mean and Ward mean, were calculated sequentially, using only the data available up to that specific point in time. This approach ensures that each row in our dataset, corresponding to a particular time point and predicting 3 days into the future from that point, uses features that are strictly based on past data, thereby preventing data leakage.

### Model Development and Validation

#### Model Training

We evaluated several machine learning approaches to find the best performing model: linear regression with lasso regularization, bagging decision trees (RandomForest), and boosted decision trees (XGBoost). These models were implemented using the sklearn and hyperopt packages in Python.

**Figure 2.**
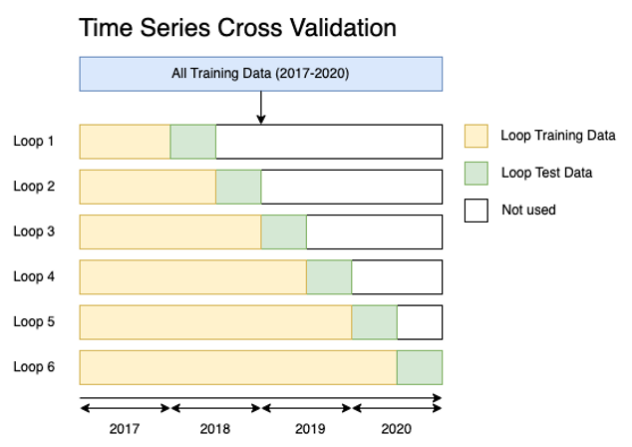
Time series cross‐validation approach used in this study. Each loop trains a model on a subset of the overall training data and tests it on the subsequent 6 months of data. With each new loop, the training set is extended, mimicking practical model usage (ie, training on historic data to predict future events) and preventing data leakage by ensuring that only past data inform each prediction.

Our dataset was split into training data (2017‐2020) and external validation data (2021). To ensure robust model training and selection, we used time series cross-validation [21]. This method of validation respects the temporal order of the data, training on past data and validating on future data, preventing information leakage and providing a reliable assessment of model generalizability (refer to Figure 2), and was chosen above the hold-out method to minimize the risk of bias from using a single train-validation data split. Within each cross-validation loop, we optimized the model parameters using Bayesian hyperparameter search. The best-performing approach was selected based on the average performance across the cross-validation loops, and this approach was then validated using the previously left-out validation dataset.

### Model Evaluation

As a baseline measure of prediction accuracy, we used the workload required for the shift at the time of the prediction. In other words, we compare the accuracy of our models’ predictions against the accuracy of the assumption that the workload required in 72 hours will be the same as the workload required for the shift at time of the prediction.

We focused on mean absolute error (MAE) and mean absolute percentage error (MAPE) as metrics for evaluating model performance, for each of which a lower value denotes better predictions. MAE is a measure of the accuracy of a model’s predictions and can be interpreted as the average error in absolute minutes, that is, a value of 180 would indicate that the predictions are, on average, 180 minutes (of nursing workload) higher or lower than the true value. MAPE is a similar metric, but expressed in absolute percentages; a MAPE value of 20% indicates that the model predictions are on average 20% higher or lower than the true value. We also recorded the improvement given by the model over the baseline, expressed as a percentage reduction in MAE when using the model versus the baseline.

When considering the potential use of such a model in practice to aid in nurse scheduling, predicting to the exact minute is arguably not the most important aspect (or indeed feasible). What is potentially more useful is the general accuracy within a certain threshold of the actual effort. For instance, we might want to know if the workload in 9 shifts (72 h) will be similar to, less than, or greater than the workload experienced in the current shift. To address this practical need, we categorized the predicted nursing workload from the model into one of three classes: (1) decreased workload: the workload 72 hours from now will be at least 360 minutes (6 h, approximately one nurse) *less* than the current shift. (2) Similar workload: the upcoming workload will be within 6 hours of the current shift workload. (3) Increased workload: the upcoming workload will be at least 360 minutes (6 h, approximately one nurse) more than the current shift.

This categorization allows for more actionable insights, helping to make more informed decisions about nurse scheduling and ensuring that staffing levels are appropriately adjusted to meet expected demands. The accuracy of the model’s predictions in this categorization was used as an extra metric on which to assess model performance. In addition, we analyzed the receiver operating characteristic (ROC) and precision-recall (PR) curves using a one-versus-rest approach for each class, as well as macro-averaged scores to provide an overall assessment across all classes. In the one-versus-rest approach, we evaluate each category individually against a grouping of the other 2 classes, allowing for an analysis of how well the model distinguishes each class. Macro-averaging was chosen because it deems all classes equally important, meaning that the resulting metric is not dominated by the class with the most instances.

#### Variable Importance

To measure the importance of each variable (feature) in predicting future workload, we calculated the permutation feature importance. This method involves randomly shuffling the values of a variable and measuring its impact on model performance, repeating the process for each variable in the dataset. A greater reduction in performance indicates higher importance, as it shows the extent to which the model relies on that variable for accurate predictions. This approach can be used independent of the model chosen and quantifies each feature’s contribution to predictive accuracy, providing interpretable insights and enhancing the explainability of the model and its predictions.

### Ethical Considerations

The cantonal ethics committee reviewed this research and determined that it did not fall under Swiss human research legislation, making it ethically permissible in this regard (Waiver number. 2021/00839 from July 27, 2021). All data records were anonymized, with patient names removed and identification numbers replaced by coded identifiers.

## Results

### Data Extraction and Transformation

The extracted LEP dataset included 39,361 hospital stays from 28,930 patients, where patients can have multiple visits and may be in multiple wards over the course of their stay. The dataset spans 5 years, 8 wards, and contains approximately 11.2 million nursing activities, with an average of just under 5 minutes per intervention. Summary statistics of the dataset can be found in Table 1. The average patient age was 56.6 years, with slightly more female patients (14,627/28,930, 50.6%); however, these figures are skewed by the maternity ward, whose patients are younger (average age 32.5) and predominantly female (6538/6733, 97.1%), the inclusion of newborn males, who sometimes receive care, means it is not 100% female.

After data transformation (refer to Figure 1), we had a dataset at the shift-level per ward, comprising 40,484 records. For each, we obtained the variables outlined in Table 2. Table 3 presents the statistics for the average nursing workload (in minutes) required for different shifts, along with the median and IQR of the number of patients per ward. The 2 neurology care wards in our dataset (Ward A and B) show intense early shift workloads, with Ward A requiring an average of 1865 minutes and Ward B an average of 1755 minutes, serving 19 and 15 patients respectively. Both intermediate care units (IMCUs)—Ward C and Ward H—maintain a relatively balanced workload across shifts, indicating consistent nursing needs in these units.

**Table 3.** Ward statistics, including the median number of patients on each ward, the average workload needed per shift (in min), as well as statistics on patient turnover.

Ward^c^	Ward type	Median (IQR) number of patients^a^	Overall average^b^ (mean) workload in minutes (SD)			Patient turnover		
		Across all shifts	Night shift (midnight – 7:59 AM)	Early shift (8 AM - 3:59 PM)	Late shift (4 PM– 11:59 PM)	Percentage of Patients still on ward 72 hours from time of prediction (%)	Mean length of stay (shifts)	Median length of stay (shifts)
Ward A	Neurology	19 (4)	838 (299)	1865 (456)	1345 (394)	51.9	15.0	10
Ward B	Neurology	15 (3)	750 (249)	1755 (480)	1292 (377)	53.0	14.8	10
Ward C	Neurology IMCU^d^	6 (2)	1043 (269)	1467 (337)	1292 (322)	51.2	12.3	5
Ward D	Urology	20 (5)	1,005 (256)	2307 (492)	1547 (412)	46.9	14.3	10
Ward E	Maternity	23 (5)	726 (208)	2168 (388)	1457 (313)	64.5	16.0	5
Ward F	Neurosurgery	19 (6)	898 (245)	2041 (455)	1494 (353)	46.7	14.3	10
Ward G	Neurosurgery	10 (3)	435 (170)	1109 (307)	873 (268)	43.5	11.6	6
Ward H	Neurosurgery IMCU^d^	6 (3)	938 (255)	1254 (327)	1092 (299)	26.6	9.9	4

a The median of the number of patients per shift, calculated across all shifts. The interquartile range (IQR) is calculated as the range between the 25th percentile (Q1) and the 75th percentile (Q3) of the data.

b The average workload is calculated over all data in our dataset for each shift.

c Actual ward names have been removed due to data privacy

d IMCU: intermediate care unit

Urology (Ward D) has the highest workload during early shifts, averaging 2307 minutes for 20 patients. Maternity (Ward E) follows closely, with a workload of 2168 minutes for 23 patients, the highest patient count across the wards. Neurosurgery wards (F, G, and H) vary, with Ward F’s early shift needing 2041 minutes for 19 patients, and Ward G’s night shift (midnight - 7:59 AM) requiring just 435 minutes on average for 10 patients, suggesting fewer overnight nursing activities.

In summary, early shifts generally place the heaviest demands on nursing staff, particularly in the Urology and Maternity wards, while night shifts typically require less nursing time. The IMCUs, however, show a relatively even workload throughout all shifts, highlighting the continuous nature of care in these specialized units.

Table 3 also shows statistics relating to the patient retention rate at each ward, showing that all wards experience significant patient turnover within the 72-hour prediction window. For example, in the neurology wards (Wards A, B, and C), on average, just over 50% of patients will still be on the ward in 72 hours (9 shifts). In many wards, less than half of the patients will remain in this same time window, and in the extreme case of Ward H, just 26.6% of patients will still be present. The maternity ward (Ward E) is the most ‘stable,’ yet still has a relatively high turnover rate.

The mean and median length of stay statistics further illustrate this turnover. In Ward H, the median stay is just 4 shifts, despite a mean stay of nearly 10 shifts, indicating that many patients leave relatively quickly. In contrast, neurology wards like Ward A and Ward B have a median stay of 10 shifts and a mean stay of 15 shifts, suggesting a slightly more stable patient presence. Nevertheless, when we compare this to the prediction window of 9 shifts, these more “stable” wards still show a high level of turnover.

### Predicting Nursing Workload

When predicting the nursing workload 72 hours/9 shifts in advance, all three models showed an improvement in prediction accuracy over the baseline, as shown in Table 4. When comparing the 2 tree-based models, random forest produced a lower MAE overall and across many individual wards, while XGBoost achieved a lower mean MAPE—a result reflecting the relative emphasis MAPE places on smaller observed values. Nevertheless, the Lasso model outperformed both Random Forest and XGBoost in overall error metrics, indicating stronger predictive capability [1].

**Table 4.** Results comparing our models to the baseline.

Ward	Ward type	Baseline MAE^a^ (MAPE^b^)	Lasso regression MAE^a^ (MAPE^b^)	Random forest MAE^a^ (MAPE^b^)	XGBoost MAE^a^ (MAPE^b^)	Lasso regression improvement over Baseline^c^ (%)
Overall	(All wards)	345.1 (28.3%)	258.8 (21.2%)	262.7 (22.0%)	270.5 (21.6%)	25.0%
Ward A	Neurology	393.0 (27.8%)	308.2 (21.4%)	329.3 (23.0%)	334.7 (22.7%)	21.6%
Ward B	Neurology	354.7 (26.0%)	279.1 (20.4%)	281.5 (20.5%)	294.0 (20.4%)	21.3%
Ward C	Neurology IMCU*^d^*	333.0 (27.4%)	258.6 (21.2%)	261.5 (21.6%)	279.4 (21.7%)	22.4%
Ward D	Urology	415.5 (28.4%)	280.3 (18.7%)	284.7 (19.4%)	299.1 (19.5%)	32.5%
Ward E	Maternity	320.3 (25.0%)	256.1 (19.2%)	237.5 (19.2%)	246.7 (19.1%)	20.0%
Ward F	Neurosurgery	365.2 (26.2%)	257.4 (18.5%)	266.5 (19.5%)	270.4 (19.1%)	29.5%
Ward G	Neurosurgery	262.3 (34.0%)	203.1 (26.1%)	210.7 (29.2%)	208.5 (26.4%)	22.6%
Ward H	Neurosurgery IMCU*^d^*	313.5 (32.2%)	225.8 (24.1%)	228.2 (24.5%)	229.2 (23.8%)	28.0%

a MAE: mean absolute error.

b MAPE: mean absolute percentage error.

c Percentage reduction in MAE of model versus baseline.

d IMCU: intermediate care unit.

The lasso regression model showed an improvement across all wards and shifts in the MAE when compared to the baseline, giving a 25.0% improvement (MAE from 345.1 min to 258.8 min). When we look at individual wards, the most substantial improvement was in Ward D, where the model MAE was 280.3 minutes, reflecting a 32.5% improvement over the baseline value of 415.5 minutes. While the predictions for the neurology and maternity wards showed a less pronounced improvement over baseline of between 20% and 23%, this still represents a significant reduction in prediction error. Lasso Regression outperformed both XGBoost and Random Forest overall, as well as across each individual ward except for just one ward, Ward E (maternity ward), where both Random Forest and XGBoost produced lower MAE values than Lasso.

**Figure 3.**
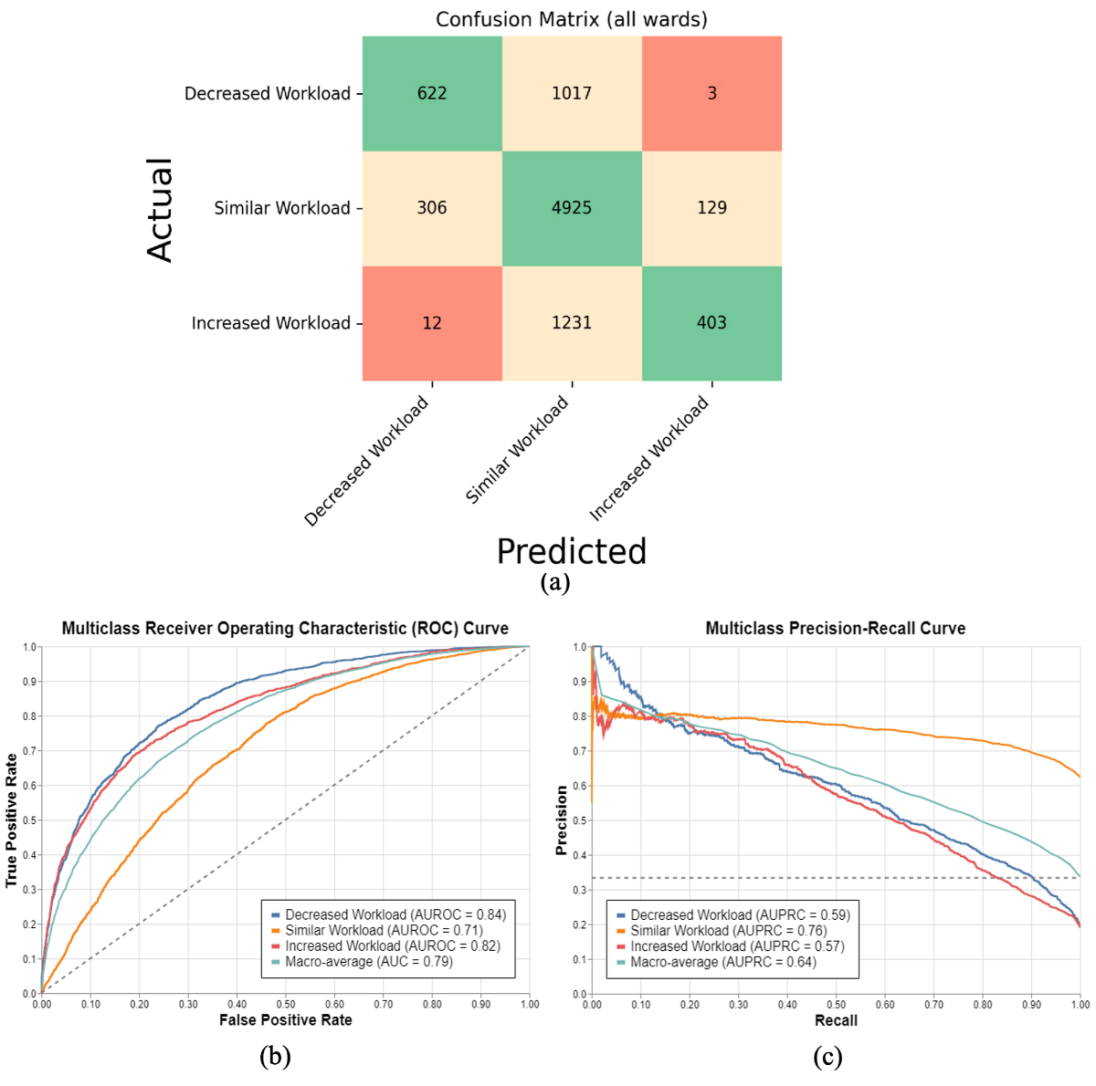
Results from categorizing future nursing workload into three groups: ‘Decreased workload,’ ‘Similar Workload’ and ‘Increased Workload.’ (**A**) shows a confusion matrix from the predictions, (**B**) multiclass receiver operating characteristic curves, and (**C**) precision-recall curves for the classification.AUC: area under the curve; AUROC: area under the receiver operating characteristic curve; AUPRC: area under the precision-recall curve.

The model’s ability to categorize the upcoming workload into “decreased,” “similar,” or “increased” (as compared to current workload levels) was also evaluated. The confusion matrix (Figure 3A) provides a summary of the model’s categorization performance, where the cell colors reflect the accuracy of the predictions: green cells along the diagonal represent correct predictions, while yellow and red cells indicate incorrect ones, with red signifying more severe errors. Precision in model predictions refers to the accuracy of the model when it predicts a specific category, whereas recall refers to the ability of the model to identify all instances of a specific category correctly. As shown in Figure 3A and Table 5, the model was correct in predicting a decreased workload 622 times, with a precision of 66.2% (662/940). Similarly, the model correctly identified 4925 instances of similar workload with a precision of 68.7% (4925/7173), and 403 increased workload instances with a precision of 75.3% (403/535). However, while the model showed a high recall of the similar workload instances (4925 out of 5360; recall of 91.9%), it achieved a lower recall in the decreased and increased workload cases, with a recall of 37.9% (622/1642) and 24.5% (403/1646), respectively. Looking at the most extreme cases of missed prediction, that is predicting a decrease when it actually increased or vice-versa, the model only made this mistake 15 (0.17%) times across the total 8648 upcoming shift predictions.

**Table 5.** Table showing the number of cases (shifts) which fall into each class of workload change, along with the model results when attempting to predict this upcoming change*.*

Class (change in workload)	Number of cases in validation data, n (%)	Model results	
		Precision	Recall
Decrease	1642 (19.0)	66.2%	37.9%
Similar	5360 (62.0)	68.7%	91.9%
Increase	1646 (19.0)	75.3%	24.5%

Figure 3B and C show the ROC and PR curves, respectively. The average AUROC of 0.79 indicates strong classification performance, with “Decreased Workload” achieving the highest AUROC (0.84) and “Similar Workload” the lowest (0.71). The PR curve, with an average AUPRC of 0.64, highlights that the model performs well when predicting “Similar Workload” (0.76), while performance is not as strong for “Increased Workload” and “Decreased Workload” (0.57 and 0.59, respectively). These differences suggest variations in class separability and PR trade-offs.

### Variable Importance From the Model

**Figure 4.**
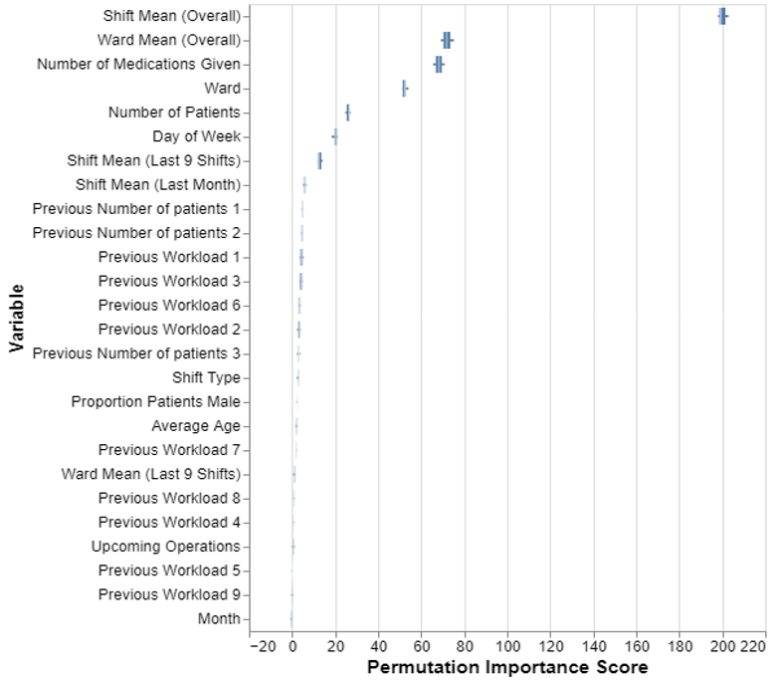
Permutation importance scores for each variable used in the lasso regression model. A higher score indicates that the variable is more important for the predictions.

The permutation importance scores (Figure 4) identify variables which the model depends on most to estimate the future nursing workload. As we can see, the predictions are based predominantly on summary information of the historical data per ward. The mean workload that was historically required for that shift type is the most “important” variable when predicting future workload, followed by the overall mean workload for that particular ward. Patient medication information, the ward, day of week, and the number of patients on the ward at time of prediction are also used by the model. However, the month variable and the specific individual values for the preceding shifts were less informative for the model.

## Discussion

### Principal Results

The goal of this study was to accurately estimate upcoming patient-related nursing workloads, using machine learning methods. The models we used consistently outperformed the baseline assumption that workload remains constant, achieving an average improvement in MAE of 25.0%.

Our descriptive analysis shows that the early shifts at the hospital consistently demand the most effort across all wards. This is especially evident in the urology (Ward D) and maternity (Ward E) wards, where high patient volumes and intense workloads highlight increased nursing needs in the early shifts. Neurological wards (Wards A and B) also face intense early shift workloads. In contrast, intensive care units (Wards C and H) maintain a steady workload across all shifts, typical of continuous care environments. Night shifts, however, generally have the lowest workload across all wards. These variations between wards highlight the need for workload estimation methods which are tailored to the specific characteristics of each ward.

The best-performing model, Lasso regression, led to a reduction in prediction error across all wards. The 25.0% improvement in MAE over the baseline demonstrates that this model provides more accurate nursing workload predictions, which is particularly important for staff planning. The model’s effectiveness is particularly evident in Ward D (urology), where MAE was reduced by 32.5% versus the baseline. This substantial improvement may be attributed to the specific characteristics of the patient population or nursing needs in this ward, indicating the model’s potential for strong performance in certain contexts. However, other wards, including neurology and maternity, also experienced notable improvements, demonstrating the model’s broad applicability and value across diverse settings. While the lasso regression model performed better overall compared to the tree-based models (Random Forest and XGBoost), it is noteworthy that the tree-based models outperformed the Lasso Regression model in certain contexts. For instance, in the maternity ward setting, both Random Forest and XGBoost achieved a lower MAE. In addition, the results show an interesting observation, where the MAPE occasionally favored one model over another (eg, for Ward H, the Random Forest MAE is better than that of XGBoost, but the opposite is true for the MAPE). These results show that the selection of the “best” model is dependent on the specific objectives and evaluation criteria of the application, emphasizing the importance of aligning metric choices with the intended use case. Future research should carefully consider these factors when designing and evaluating predictive models for use in practice.

The model effectively categorizes upcoming workload changes as “decreased,” “similar,” or “increased,” showing potential in aiding staffing decisions. Overall, the ROC-AUC and PR-AUC results show strong performance, and the model achieves solid precision scores, with high recall for a ‘similar’ upcoming workload. Crucially, the model minimizes critical errors, that is, incorrectly predicting an increase or decrease in workload when the opposite happens in reality, showing a strong potential for application in staff scheduling.

The variable importance results (Figure 4) show that the model predominantly uses historical averages of nursing workload to predict future values. These variables prove to be the most important for predicting future workloads, understandably suggesting that past patterns are a reliable indicator of future demands. The high importance of these variables highlights the need to carefully capture and analyze historical data to enable accurate predictions. In addition to historical trend data, the model also uses information about patient medication, the day of the week, and the number of patients on the ward at the time of the prediction. These variables provide additional contextual information that allows the model to better understand the specific conditions of the ward, while also suggesting that with an expanded feature set which includes more patient-specific data, we could expect the model’s performance to further improve. Interestingly, the month and individual values from recent shifts are less informative, suggesting that seasonal fluctuations and short-term changes have a smaller impact on workload than ward-specific factors and long-term patterns.

In summary, this study demonstrates that machine learning models can help improve workload prediction accuracy in healthcare settings, potentially leading to better staffing decisions and enhanced patient care. Although we used relatively basic machine learning methods, primarily linear regression and tree-based models, while also simplifying the temporal aspect of the data by using summary statistics (rather than incorporating the full time-series information into the models), our approach yielded promising results. In its present form, the model reliably predicts routine shifts but can struggle with sudden changes in nursing workload levels. However, the strong precision means that when it does predict a change, it is usually correct, therefore making it more suitable as a decision-support tool rather than the sole determinant of staffing levels. More advanced time series methods, combined with a broader range of data sources—such as planned patient arrivals, departures, and patient-specific health data—should enhance model accuracy and adaptability, thus improving the case for such methods for use in real-world applications. This study serves as an introductory step, demonstrating the potential of machine learning in workload prediction and paving the way for future research into more advanced and adaptive modeling approaches.

### Limitations

During the study’s planning phase, we found no other publications with similar objectives of applying machine learning in such a broad context, highlighting the innovative nature of our work but also leading to a limited ability to compare our results with external studies.

One significant limitation of this study is the quality of the LEP data. While theoretically, all patient care provided by nurses is recorded, in reality, this is often not the case due to factors such as high workload, lack of time, and incomplete adherence to documentation protocols [15,16]. It is also conceivable that completeness of LEP data varies across different wards in the hospital, for example, due to training levels and time constraints, but also potentially due to the ‘culture’ in that ward, and the emphasis that is placed on completeness of recorded data. These discrepancies suggest that our models are predicting recorded workload rather than the actual required workload. For this paper, we assumed that the recorded workload reflects the actual workload, but this assumption needs to be tested before practical application.

The range of modeling techniques which we could explore in this phase of the study was limited due to the low level of computing power available, preventing the use of neural networks. While this choice was appropriate due to these constraints, future work would benefit from exploring more complex models which may improve prediction accuracy.

Another limitation comes from the uncertainty of predicting several days ahead. In a hospital setting, a 3-day prediction window is considerable, as patient turnover, emergency surgeries, fluctuations in patient condition, among other factors, can all affect nursing care requirements, and these unpredictable events are challenging—if not impossible—to fully capture in our dataset. While a shorter prediction window would mitigate this issue, it would also reduce the predictions’ usefulness by leaving less time for nurse scheduling adjustments. Future work could be carried out to find the optimum prediction window so as to lessen the inherent uncertainty in future predictions, while also providing enough time for the predictions to be useful.

In this project, the concept of ‘data drift’ was not fully addressed. This refers to potential changes in hospital methods and healthcare practices over time, which can alter patient characteristics and overall workload. Our models were trained on data from 2017‐2020 and tested on data from 2021, indicating that they may already be outdated. To maintain the effectiveness of the model in future implementation phases, it will be essential to incorporate updated data regularly, continuously retrain the model, and closely monitor its performance over time to ensure it remains reliable.

Another limitation of this study is that we did not consider the number of nurses on a ward or their experience levels, both of which could influence the recorded workload. While we cannot include the future staffing level in the model (as this is unknown at the time of prediction), we could possibly devise and predict nurse number-neutral workload statistics, such as dividing the recorded workload by the number of nurses. In addition, we could develop a counterfactual model that predicts future workload under various staffing scenarios, including different nurse numbers and varying levels of education and experience among the nursing staff. Such models could be key to ensuring a high quality of nursing care: optimizing nurse scheduling should not lead to understaffing where just the bare minimum gets performed, leading to compromised care quality and nurse satisfaction.

Finally, this study did not account for the complexity of nursing care—tasks of the same duration can vary greatly in complexity. The concept of “nursing care complexity” complements “nursing workload,” and together, these 2 concepts define “patient acuity.” The complexity of nursing care is a multifaceted and dynamic process, characterized by instability, uncertainty, and variability throughout various stages of care [22]. Carr-Hill et al [10] highlight the importance of considering both objective elements (such as time spent on tasks) and subjective factors (such as perceived stress) when assessing workload. Therefore, predicting nursing care complexity alongside recorded workload could improve scheduling by allowing adjustments based on both the quantity and complexity of nursing tasks as well as the subjective burden of nursing personnel, leading to more effective nurse allocations and higher nurse satisfaction.

### Future Work

While our findings are promising, they also highlight key avenues for further development. To optimize this approach, model refinement is crucial. Incorporating more diverse data sources, exploring tailored time-series modeling techniques, and considering additional data points could significantly enhance prediction accuracy. Future research should also be conducted into finding out the extent of the discrepancy between workload recorded in LEP and the actual workload.

In this study, we used aggregated shift-level data for predicting future patient nursing requirements. Another option would be to model nursing requirements at the patient level and to sum these to get a prediction for the ward. This approach would offer more detailed results, but the high rate of change in patient make-up (refer to Table 3) would make getting accurate predictions difficult. This avenue could be explored in future work, especially if data relating to planned patient departures and arrivals were available.

Future work could also benefit from the use of more advanced models, such as neural networks, which could allow for more flexible pattern recognition and ultimately improve prediction accuracy. In addition, the seniority or experience level of nursing staff may influence how much work is carried out; for example, more experienced nurses may work more efficiently or undertake slightly different types of patient care. Including information relating to the nursing staff composition could therefore improve future model performance. However, it would be important to consider how such models, if used in practice, would influence staffing decisions – both the number and experience of staff assigned to a shift. Such changes in staffing composition could lead to a feedback loop, where workload predictions influence staffing decisions, thereby influencing future workload levels, and subsequently future predictions. One potential way to mitigate such effects would be to regularly update the model with up-to-date data to help reflect any changes in staff planning practices, which were driven by the model itself and would be reflected in the data.

The practical benefits of the approach outlined in this paper remain to be fully demonstrated. Controlled trials in real-world hospital settings are necessary to evaluate whether machine-learning-powered workload prediction translates into an improvement in staff planning, especially when compared to professional judgment alone.

### Conclusions

This study demonstrates the potential of applying machine learning to nursing workload data for predicting future nursing workloads, showing an improvement in prediction accuracy over the baseline. It promises to greatly help improve the efficiency of nurse scheduling and staff satisfaction. Future work should focus on addressing data quality concerns, exploring more advanced modeling techniques, and validating these techniques through real-world trials. In addition, exploring the integration of such predictive models into existing nurse scheduling workflows and evaluating their impact on operational efficiency and staff satisfaction in real-time settings would further enhance the value of these methods.

## Supplementary material

10.2196/66667 Multimedia Appendix 1 Literature review.

## Abbreviations

EHR: electronic health records
IMCU: intermediate care unit
KISIM: Klinikinformationssystem
LEP: Leistungserfassung in der Pflege; documentation of nursing activities
MAE: mean absolute error
MAPE: mean absolute percentage error
PR: precision-recall
ROC: receiver operating characteristic
