# Supplementary material for: Development of a Data-Based Method for Predicting Nursing Workload in an Acute Care Hospital: Methodological Study
Source: J Med Internet Res. 2025 Sep 16;27:e66667. doi: 10.2196/66667 (PMC12440230; doi:10.2196/66667)
Supplement: Multimedia Appendix 1 [file jmir-v27-e66667-s001.docx]

| **Year** | **Study** | **Author(s)** | **Focus** | **Limitations** | **Relevance to Our Study** |
| --- | --- | --- | --- | --- | --- |
| 2020 | Machine Learning in Optimizing Nursing Care Delivery Models | Bergmeir & Benítez | Compares different ML techniques for workload prediction | Focuses on methodology rather than implementation | Demonstrates ML potential but lacks real-world application |
| 2021 | Nursing Workload: Use of Artificial Intelligence to Develop a Classifier Model | Hasselgård et al. | Classifies patient workload levels using AI | Does not predict future workload, only categorizes current levels | Useful for workload assessment but lacks forecasting capability |
| 2021 | Staff Management with AI: Predicting the Nursing Workload | Harper et al. | Identifies predictors of workload using AI techniques | Specialist-focused, lacks shift-based workload forecasting | Supports predictor identification but not full workload forecasting |
| 2023 | Estimation of Clinical Workload and Patient Activity Using Deep Learning | Pashaei et al. | Uses optical flow and deep learning to estimate clinical workload | Does not predict future workload; estimates real-time activity | Demonstrates alternative data sources but lacks shift-level forecasting capabilities |
| 2023 | Use of a Multiscale Vision Transformer to Predict Nursing Activities Score | Ranegger & Baumberger | Video-based estimation of nursing workload using computer vision | Focuses on image-based workload estimation, not shift-level forecasting | Novel data collection method but not directly applicable to workload forecasting |
| 2024 | Predicting Nursing Workload in Digestive Wards Based on Machine Learning | Song et al. | Predicts patient-specific workload dynamically over hospitalization | Limited to a single specialty ward and does not model patient discharge or transfers | Supports patient-level prediction but lacks generalizability to shift-level planning |
| 2024 | Nurses' Workload Prediction in Hospitals: A Machine Learning-Based Approach | Unknown (HAL Repository) | Explores ML potential for workload prediction | Discusses methodology but does not implement a predictive model | Highlights need for a validated, shift-level forecasting model |
| 2024 | Nursing Workload Prediction for Upcoming Shifts: A Retrospective Observational Exploratory Study in the Postoperative and Intensive Care Unit | Hasselgård et al. | Uses the Nursing Activities Score (NAS) to predict workload for the upcoming shift in a postoperative and ICU setting | Limited to a single ICU, relies solely on NAS without incorporating broader workload factors, and predicts only shift-to-shift workload rather than long-term forecasting. | Demonstrates feasibility of workload prediction but lacks the broader scope, multi-ward data, and machine learning optimization used in our study to predict workload up to 72 hours in advance. |
